# Spontaneous regression of breast cancer with immune response: a case report

**DOI:** 10.1186/s40792-020-01103-5

**Published:** 2021-01-06

**Authors:** Masahiro Ohara, Yumiko Koi, Tatsunari Sasada, Keiko Kajitani, Seishi Mizuno, Ai Takata, Atsuko Okamoto, Ikuko Nagata, Mie Sumita, Kaita Imachi, Mayumi Watanabe, Yutaka Daimaru, Shingo Kawamura

**Affiliations:** 1 Department of Breast Surgery, Hiroshima General Hospital, 1-3-3 Jigozen, Hatsukaichi, Hiroshima 738-8503 Japan; 2 Section of Pathological Research and Laboratory, Hiroshima General Hospital, 1-3-3 Jigozen, Hatsukaichi, Hiroshima 738-8503 Japan; 3 Suzumine Imanaka Clinic, 4-2-31, Inokuchi, Nishi-ku, Hatsukaichi, Hiroshima 733-0842 Japan; 4 Department of Breast Oncology, National Hospital Organization Kyushu Cancer Center, 3-1-1 Notame, Minami-ku, Fukuoka, 811-1395 Japan

**Keywords:** Breast cancer, Spontaneous regression, Immunogenic cell death

## Abstract

**Background:**

Spontaneous regression (SR) is a rare phenomenon in which a cancer disappears or remits without treatment. We report a case of breast cancer that showed spontaneous tumor regression in the surgical specimen after core needle biopsy.

**Case presentation:**

A 59-year-old woman came to our hospital complaining of a painful lump in the right breast. In the upper-outer quadrant of the right breast, a tumor with an unclear boundary, 30 mm in diameter, was palpable. In pathological findings from needle biopsy, the tumor was diagnosed as solid-type invasive ductal breast carcinoma. Partial coagulation necrosis was generated in estrogen receptor-negative, HER2-negative, and AE1/AE3-positive ductal carcinoma without infiltration of lymphocytes. Surgery for right breast cancer was then performed. Histological examination of the surgical specimen revealed the tumor was invasive ductal carcinoma with lymphocyte infiltration, coagulation necrosis, and fibrous tissue with hemosiderin. The tumor formed a solid nest, 3 mm in diameter, suggesting the possibility of SR.

**Conclusions:**

Immune responses, infection, hormones, surgical stress, and ischemia have been reported as mechanisms of SR. The findings in this case strongly suggest that SR of breast cancer is associated with anti-tumor immune responses.

## Background

Spontaneous regression (SR) of cancer is a rare but well-documented biological phenomenon. SR is defined as “the partial or complete disappearance of a tumor in the absence of any treatment capable of regression” [1, 2]. Breast cancer regression was reported in 43/741 cases of spontaneously regressing cancers compiled and summarized by Challis and Stam in a review of the period from 1900 to 1987 [3], and few additional reports have been published since then [4–9]. Various mechanisms are considered to be associated with this phenomenon, including immune mediation, tumor inhibition by growth factors and/or cytokines, induction of differentiation, hormonal mediation, and tumor necrosis.

Spontaneously induced T-cell-mediated immunological responses have recently gained attention in multidisciplinary cancer treatment, since more than 30% of durable clinical responses including complete response are observed just with administration of antibody to block the PD-1/PD-L1 inhibitory immunological checkpoint signal in various cancer patients [10, 11]. Spontaneously induced immunological responses could thus also be an important mechanism in the SR of cancer.

We report herein a case of SR of breast cancer with induced immune responses. Immunohistochemically, we confirmed that partial coagulation necrosis was generated in estrogen receptor-negative, HER2-negative, and AE1/AE3-positive ductal carcinoma without infiltration of lymphocytes on preoperative pathological findings. Postoperative histopathological findings consequently showed that most tumor cells had been replaced by granulation tissue and residual ductal carcinoma had been driven into a smaller area by the infiltration of lymphocytes, suggesting that the SR of this breast cancer could be due to anti-tumor immune responses induced by unexplained inflammation.

## Case presentation

**Fig. 1:**
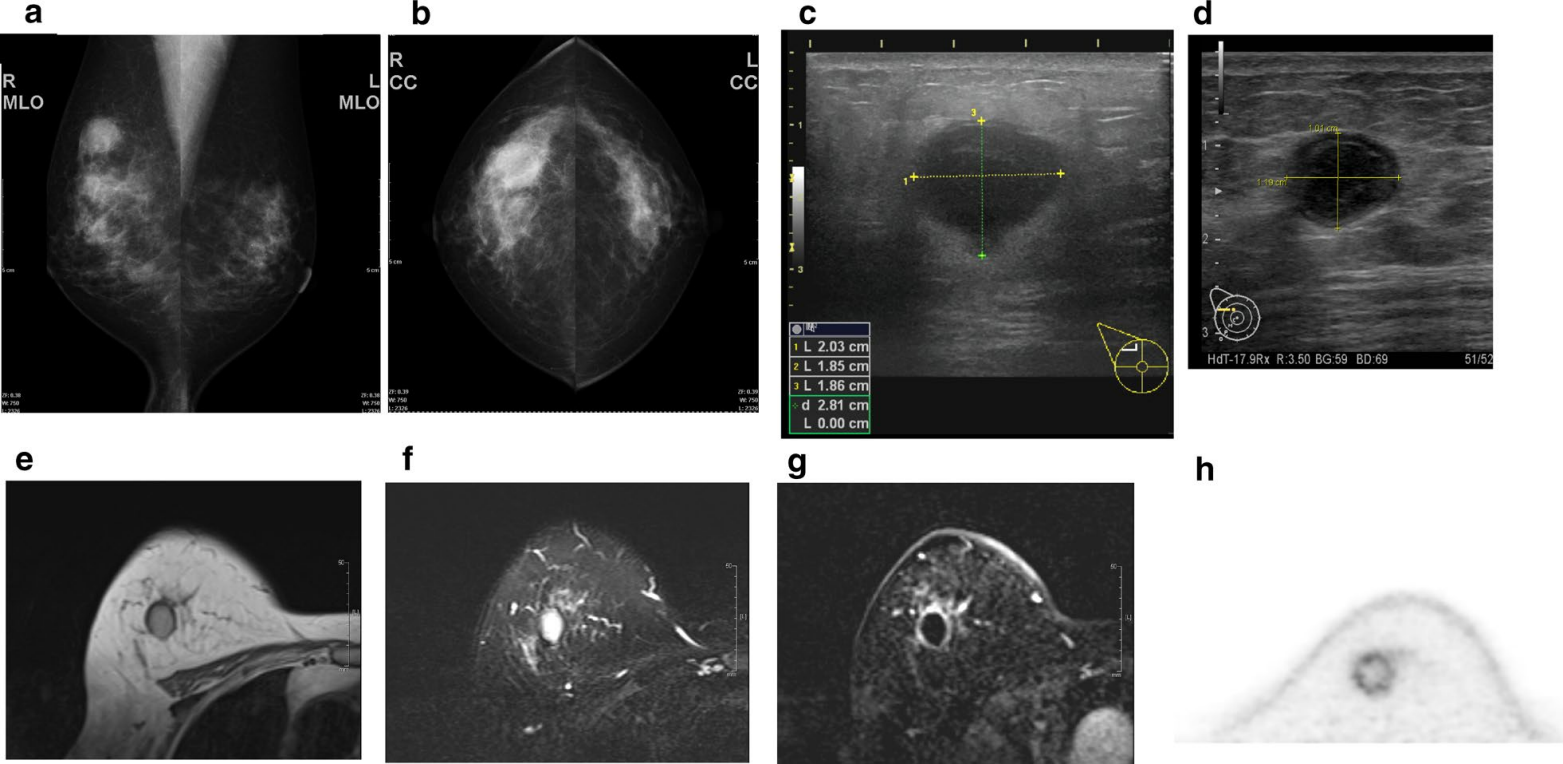
Preoperative imaging findings. **a** Mediolateral oblique-view mammogram. **b** Craniocaudal-view mammogram. A mass with a clear boundary is recognized in the right breast. **c** Ultrasonogram at first visit. A smooth, round mass is apparent in the upper-outer quadrant of the right breast. Subcutaneous fat tissue around the tumor appears as a highly echogenic, edematous layer. **d** Ultrasonogram just before surgery. Edema around the tumor has disappeared. **e** T1-weighted magnetic resonance imaging (MRI). **f** T2-weighted MRI. **g** MRI with early gadolinium enhancement A smooth, round mass with slight hyperintensity on T1-weighted MRI and hyperintensity on T2-weighted MRI. Only the marginal region of the tumor appears enhanced. **h** Ring-type dedicated breast positron emission tomography. A ring-shaped accumulation of ^18^F-fluorodeoxyglucose is evident in the right breast

**Fig. 2:**
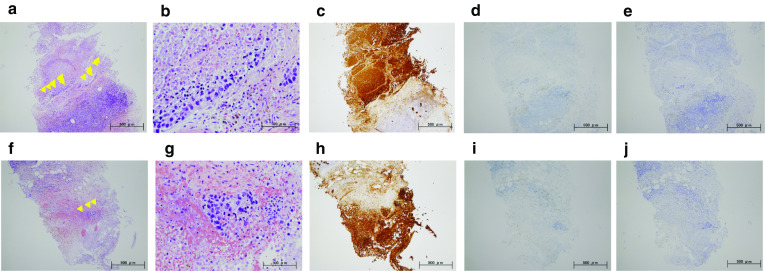
Preoperative pathorological findings. The histopathological findings of the right breast from core needle biopsy (**a**, **f**: hematoxylin and eosin (HE) × 40; **b**, **g**: HE × 200). Immunohistochemistry study for AE1/AE3, estrogen receptor (ER), and HER2 (**c**, **h**: AE1/AE3 × 40; **d**, **i**: ER × 40; **e**, **j**: HER2 × 40). Small foci of atypical ductal cells are recognized (yellow arrow). Partial coagulation necrosis is generated in estrogen receptor-negative, HER2-negative, and AE1/AE3-positive ductal carcinoma without infiltration of lymphocytes

A 59-year-old woman came to our hospital with a chief complaint of a painful lump in the right breast. She regularly visited her primary doctor for type 2 diabetes, hypertension, and hyperlipidemia. She was treated with metformin, olmesartan medoxomil/ azelnidipine, and pravastatin for less than 5 years. She had no family history associated with breast cancer. Reviewing her past history, she had received total hysterectomy at age of 47 for a uterine leiomyoma. A tumor with an unclear boundary was palpable in the upper-outer region of the right breast, about 30 mm in diameter along the major axis. Mammography revealed a mass with a clear boundary, 19 × 18 mm in size, in the middle outer portion of the right breast (Fig. 1a, b). Ultrasonography revealed a smooth, round mass measuring 20 × 18 × 18 mm in size, in the upper-outer quadrant of the right breast. Subcutaneous fat tissue around the tumor appeared as a highly echogenic, edematous region (Fig. 1c). In pathological findings from needle biopsy, the tumor was diagnosed as solid-type invasive ductal breast carcinoma. Partial coagulation necrosis was generated in estrogen receptor-negative, HER2-negative, and AE1/AE3-positive ductal carcinoma without infiltration of lymphocytes (Fig. 2a–j, 3a–e). Thirteen days after core needle biopsy, magnetic resonance imaging (MRI) was performed. MRI showed a smooth, round mass measuring 20 × 16 × 15 mm in size with slight hyperintensity on T1-weighted MRI and with high intensity on T2-weighted MRI. Only the marginal region of the tumor was enhanced (Fig. 1e–g). Ring-type dedicated breast positron emission tomography showed a ring-shaped fluorodeoxyglucose accumulation in the right breast (Fig. 1h). We performed ultrasonography just before surgery, showing that the tumor remained the same shape as before, although the size had decreased to 12 × 10 × 11 mm and the edema around the tumor had disappeared (Fig. 1d). Right partial breast resection and sentinel lymph node biopsy was performed 53 days after core needle biopsy. On histological examination of the surgical specimen, the tumor was showed lymphocyte infiltration, coagulation necrosis, and fibrous tissue spread with hemosiderin deposition. The tumor formed a solid nest, 3 mm in diameter, suggesting the possibility of SR (Fig. 4a–d). A significant aggregation of lymphocytes was observed around tumor cells. These lymphocytes comprised CD3-positive, CD4-positive, or CD8-positive T cells (Fig. 5a–c) accompanied by aggregations of CD20-positive B-cells (Fig. 5d), but few CD56-positive natural killer (NK) cells (Fig. 5e). In addition, residual tumor cells in the surgical specimen did not express PD-L1 (Fig. 5f).

**Fig. 3:**
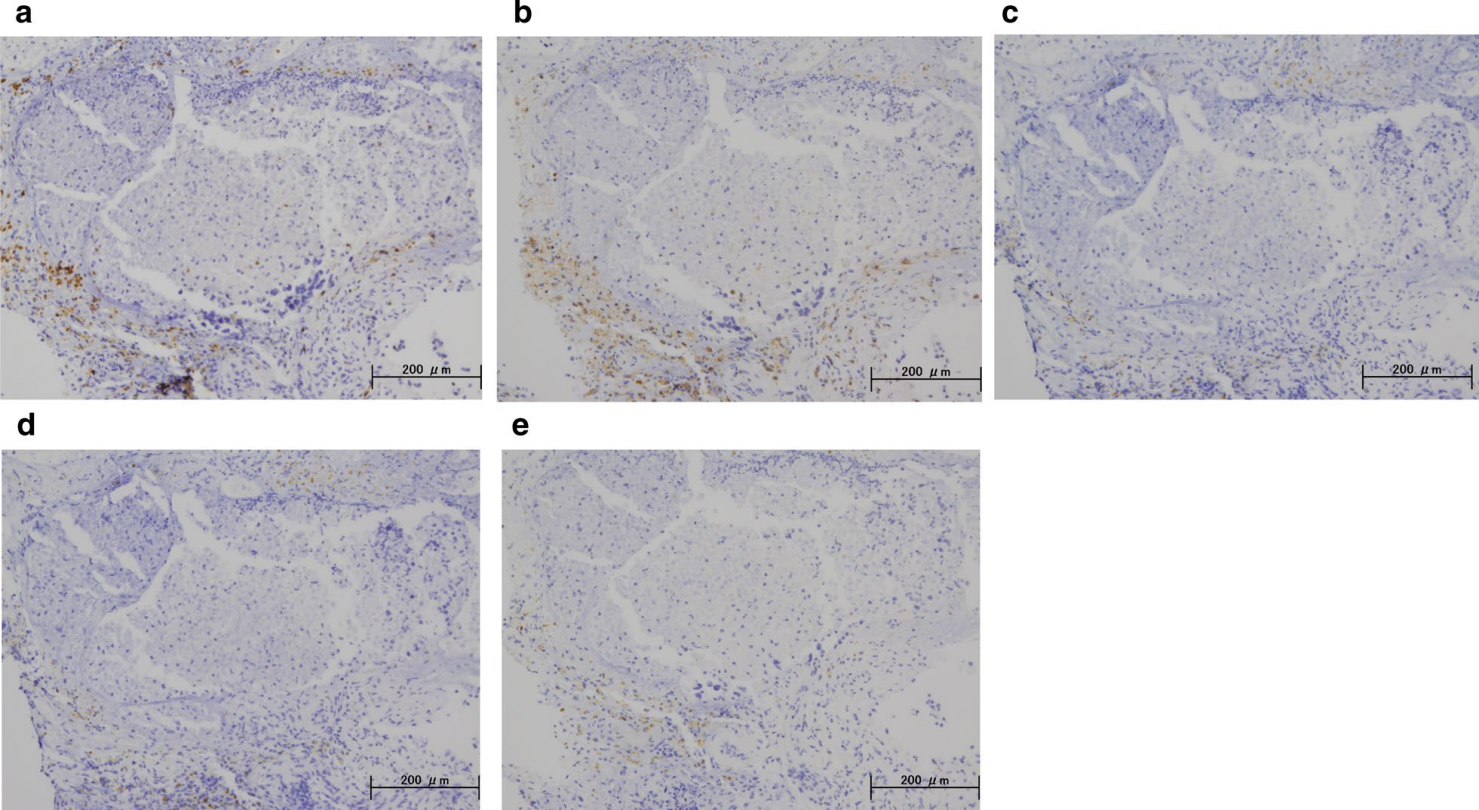
Immunohistochemical staining for core needle biopsy specimens. Immunohistochemistry study for immunological surface markers (**a** CD3; **b** CD4; **c** CD8; **d** CD20; **e** CD56. All original magnifications are × 200). The necrotic area in needle biopsy did not contain immune cells

**Fig. 4:**
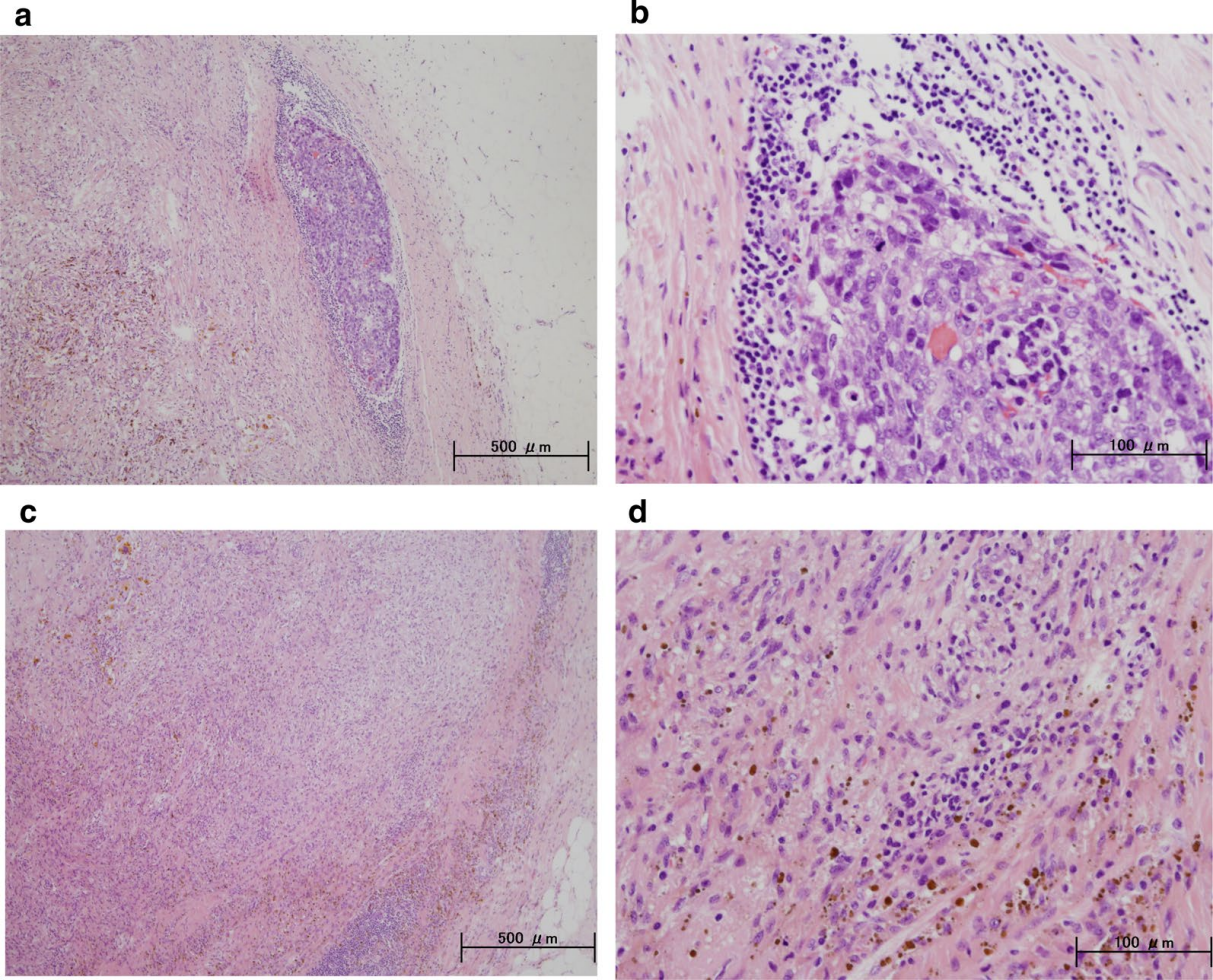
Postoperative histopathological findings. Histopathological findings of the resected breast tissue (**a**, **c**: hematoxylin and eosin (HE) × 40; **b**, **d**: HE × 200). The tumor shows lymphocyte infiltration, coagulation necrosis, and fibrous tissue with hemosiderin deposition

**Fig. 5:**
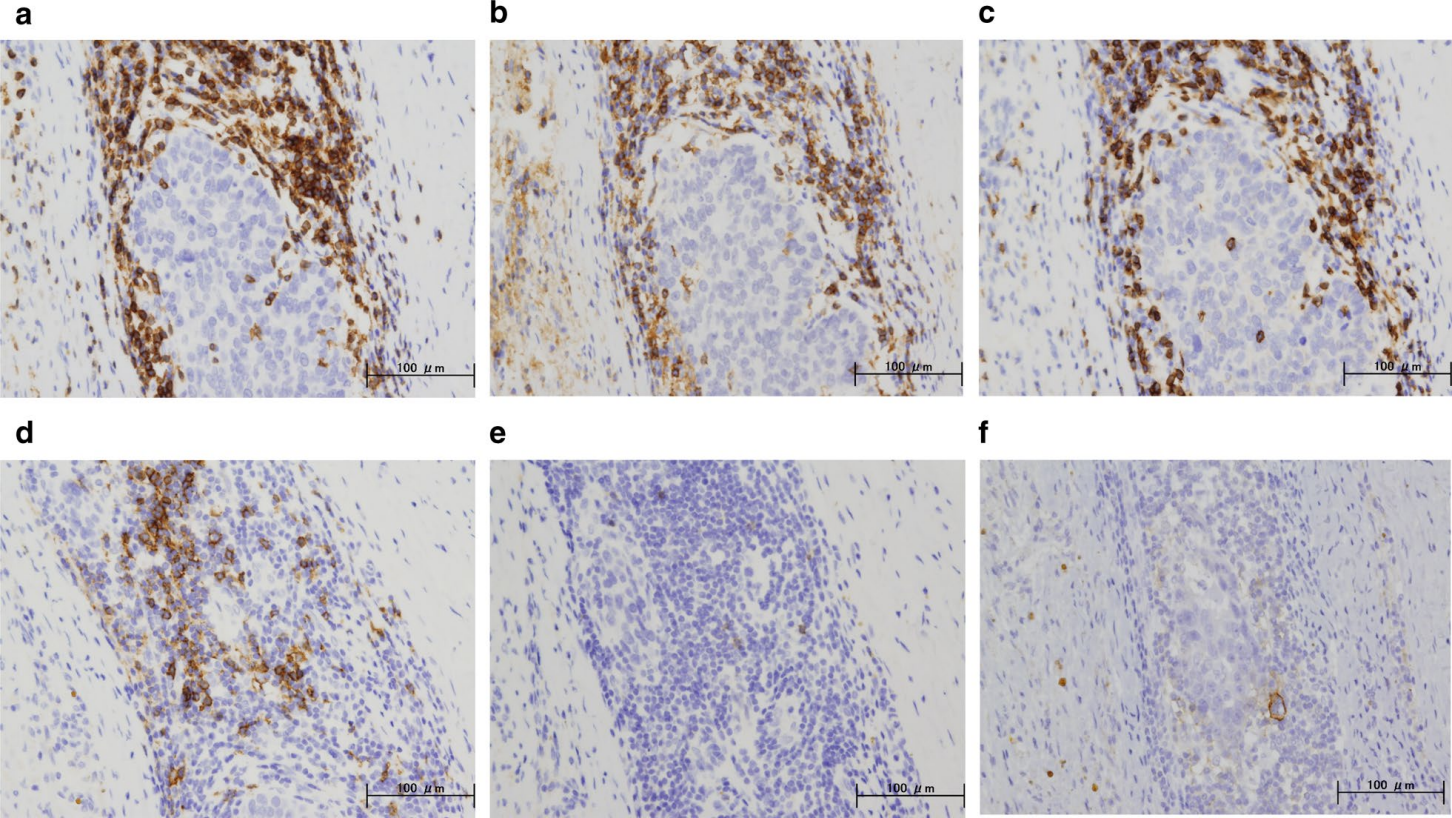
Immunohistochemical staining for the primary breast cancer. Immunohistochemistry study for immunological surface markers (**a** CD3; **b** CD4; **c** CD8; **d** CD20; **e** CD56; **f** PD-L1. All original magnifications are × 200). Immunohistochemical staining for the primary breast cancer. CD3-positive (**a**), CD4-positive (**b**), and CD8-positive T-cells (**c**), aggregation of CD20-positive B-cells (**d**), and occasional CD56-positive NK cells are detected (**e**). Ductal carcinoma cells are immunonegative for PD-L1 (**f**)

Adjuvant radiation therapy (50 Gy in 25 fractions) to the whole breast was performed. The patient is being followed-up closely, with examinations every 3–4 months, and is undergoing regular breast examinations, breast ultrasonography, mammography and tumor marker evaluations (carcinoma antigen 15–3 and carcinoembryonic antigen). After 16 months of follow-up, we have not observed any signs of cancer relapse, and the patient has remained free of the disease.

## Discussion

SR of breast cancer is a rare event that is recognized in the medical literature, but is still an unexpected phenomenon. Due to the rarity of SR, case reports and studies of the reported single cases remain restricted by the lack of sufficient data on a number of biological behaviors and their clinical significance.

Possible mechanisms underlying spontaneous cancer regression include immune system or hormonal mediation, tumor inhibition by growth factors/cytokines, induction of differentiation, elimination of a carcinogen, tumor necrosis, angiogenesis inhibition, psychological factors, apoptosis, and epigenetic mechanisms [1, 2]. This phenomenon has been speculated to be possibly related to trauma or infection [1, 4]. In the current case, the patient could not remember any traumatic or infectious events involving the site. Furthermore, she did not change her pattern of living and her medication regimen was not changed before surgery. Although the patient took metformin and pravastatin for 5 years, the possibility could not be denied that metformin and pravastatin has played an important role in this regression. Retrospective studies have demonstrated that metformin and statin decreased incidence and recurrence rate of breast cancer potentially [12–15]. Pain symptoms at the tumor site and the edema around the tumor on ultrasonography showed the existence of inflammation, irrespective of cause. Preoperative histopathological findings revealed tumor generation and necrosis without infiltration of inflammatory cells at that time.

Immunogenic cell death (ICD), a newly defined form of cell death, may involve recruitment of the host immune system, thereby resulting in immune memory and advantageous systemic effects. ICD of cancer cells can induce effective antitumor immune responses through activation of dendritic cells (DCs) and consequent activation of specific T-cell responses [16, 17]. ICD is defined as several steps resulting in the translocation of calreticulin to the cell surface (an “eat-me” signal for DCs) and the release of danger signals such as HMGB1 and ATP, which are essential for the promotion of CD8 T-cell anticancer responses [18]. ICD can be induced by chemotherapeutic agents such as anthracyclines and oxaliplatin, or radiotherapy and photodynamic therapy, or some physical therapies [19]. In the present case, surgical specimens showed tumor cells surrounded by abundant lymphocytes, while core needle biopsy specimens had not contained tumor-infiltrating lymphocytes. Unknown ICD-derived anti-tumor immunity was speculated to have caused residual tumor regression.

The roles and subsets of tumor-infiltrating lymphocytes have been discussed in cases involving SR of breast cancer [5, 6]. Both CD4- and CD8-positive subsets of CD3-positive T-cells have been implicated in the genesis of SR [6], although NK cells were suggested in another case [5]. In the present case, the aggregated cells were mainly CD3-, CD8-, or CD4-positive T-cells, while CD56-positive NK cells were not observed, consistent with a previous report [6]. A few reports have discussed the triggers generating antitumor immunity during spontaneous tumor regression. The initiating event might be related to the trauma from biopsy, as suggested by Maillet et al. [5]. To the best of our knowledge, this is the first case to suggest that ICD of tumor cells could induce anti-tumor immunity resulting in SR of breast cancer based on pre- and postoperative pathological findings. Furthermore, the immune response could potentially have continued and the patient might have achieved complete remission without surgery, as the final tumor cells did not express PD-L1 despite the infiltration of abundant lymphocytes.

## Conclusions

Our case distinctly indicates that SR of breast cancer is associated with ICD. One limitation of our study was that we could not show concrete reasons and molecular markers for ICD. Recognizing the presence of SR and ICD of breast cancer is important, and a more detailed understanding of the mechanisms underlying SR and ICD would provide significant implications for cancer prevention and therapeutics.

## Data Availability

All data analyzed during this study are included within the manuscript. The datasets used and/or analyzed during this study are available from the first author on reasonable request.

## Abbreviations

SR: Spontaneous regression
ICD: Immunogenic cell death
DCs: Dendritic cells
MRI: Magnetic resonance imaging
HE: Hematoxylin and eosin
